# CNS Macrophages and Infant Infections

**DOI:** 10.3389/fimmu.2020.02123

**Published:** 2020-09-18

**Authors:** Alexander Oschwald, Philippe Petry, Katrin Kierdorf, Daniel Erny

**Affiliations:** ^1^Faculty of Medicine, Institute of Neuropathology, University of Freiburg, Freiburg, Germany; ^2^Faculty of Biology, University of Freiburg, Freiburg, Germany; ^3^CIBBS Centre for Integrative Biological Signalling Studies, University of Freiburg, Freiburg, Germany; ^4^Center for Basics in NeuroModulation (NeuroModulBasics), Faculty of Medicine, University of Freiburg, Freiburg, Germany

**Keywords:** microglia, CNS-associated macrophages, prenatal infections, postnatal infections, TORCH, maternal immune activation

## Abstract

The central nervous system (CNS) harbors its own immune system composed of microglia in the parenchyma and CNS-associated macrophages (CAMs) in the perivascular space, leptomeninges, dura mater, and choroid plexus. Recent advances in understanding the CNS resident immune cells gave new insights into development, maturation and function of its immune guard. Microglia and CAMs undergo essential steps of differentiation and maturation triggered by environmental factors as well as intrinsic transcriptional programs throughout embryonic and postnatal development. These shaping steps allow the macrophages to adapt to their specific physiological function as first line of defense of the CNS and its interfaces. During infancy, the CNS might be targeted by a plethora of different pathogens which can cause severe tissue damage with potentially long reaching defects. Therefore, an efficient immune response of infant CNS macrophages is required even at these early stages to clear the infections but may also lead to detrimental consequences for the developing CNS. Here, we highlight the recent knowledge of the infant CNS immune system during embryonic and postnatal infections and the consequences for the developing CNS.

## Introduction

During fetal and postnatal development the central nervous system (CNS) is constantly rearranged to construct and elaborate neuronal circuits needed to fulfill complex neuronal tasks later in life (1). Even though the CNS is supposed to be immune privileged to a certain degree (2, 3), infant infections are able to reach the CNS and can cause immunopathologies, severe long-term sequelae of the CNS or even death. In 2015, 5.9 million children below the age of 5 died due to different circumstances including preterm birth complications, but also malnutrition and infections (4–6). Roughly half of these deaths are caused by infectious diseases such as pneumonia, diarrhea, neonatal sepsis and malaria, mostly in low- and middle-income countries (7, 8). During the last two decades, the numbers of child mortality due to infections were dramatically reduced by improvements in hygienic standards, vaccination programs and introduction of new antimicrobial drugs (9). However, there are still millions of children worldwide reported with prenatal and postnatal infections affecting the CNS and causing CNS pathologies (10). Therefore, fetal and early infant stages until the first years of life seem to present a vulnerable window where infections reaching the CNS can cause detrimental pathologies and malformations.

The CNS is one of the earliest formed organs during embryogenesis and needs immune cell coverage early on to assure correct CNS development and immune defense against pathogens (11). Therefore, macrophages appear in the brain very early during development. Microglia and CNS-associated macrophages (CAMs) are the tissue resident macrophages of the brain, the former residing in the CNS parenchyma and the latter inhabiting the CNS interfaces such as the perivascular space, the meninges and the choroid plexus (12–14). In mice, it was shown that microglia derive from erythro-myeloid progenitors (EMP) from the yolk sac and start to colonize the brain parenchyma at embryonic day (E) 9.5 (12, 13, 15–17). As soon as the progenitors enter the tissue they extensively expand by proliferation, distribute throughout all brain regions during development and differentiate into mature microglia (12, 18). Interestingly, microglia are not exchanged by hematopoietic stem cell (HSC)-derived circulating progenitors during later development and adulthood during steady state (19–21). Even though the HSC-independent development and endogenous maintenance is assumed for mammals during physiological conditions, it was shown in other species such as the zebrafish that microglia can be derived from different hematopoietic origins during development (22, 23). However, upon neuroinflammation in the mammalian CNS, for example during infections, recruitment of monocyte-derived macrophages is widely described due to release of chemokines and cytokines in the parenchyma and opening of the blood-brain barrier (BBB) (24).

Similar to microglia, most CAM populations also arise from EMP-derived macrophage progenitors in mammals (14, 25). However, it has been shown that subpopulations of the choroid plexus macrophages and meningeal macrophages, namely stromal and dural macrophages, are partially replaced by bone marrow-derived circulating monocytes during adulthood (14, 26). In mice it was described that CAMs start to colonize their specific niche in the CNS interfaces from E12.5 when anatomical structures of the brain interface start developing (14). However, the exact timing and distribution of the cells in the developing interfaces during development is ill-defined. In humans, there is only limited data available on the development of CNS macrophages. For microglial progenitors, it was described that they colonize the developing neuroectoderm starting around gestational week 4.5 (27–29). Post-mortem studies indicate that ionized calcium binding adaptor molecule 1 (Iba-1)^+^ microglial progenitors enter the brain via the meninges, choroid plexus and ventricular zone (28, 29). Human CAMs are detected shortly after in the developing CNS interfaces. Macrophages in the meninges of the human optic nerve were described as early as gestational week 8 (30). Another study describes first stromal choroid plexus macrophages around gestational week 11 (31). Future studies are needed to explore the development of human CNS macrophages in more depth.

CNS macrophage differentiation is a highly dynamic process during pre- and postnatal development controlled by both an intrinsic genetic program and extrinsic factors and is essential for CNS tissue homeostasis. Microglia differentiation depends on the transcription factors spleen focus forming virus (SFFV) proviral integration oncogene (*Sfpi*, encoding PU.1), interferon regulatory factor 8 (*Irf8*) and spalt like trancription factor 1 (*Sall1)* while independent of cellular myeloblastosis oncogene (*c-Myb*), inhibitor of DNA-binding 2 (*Id2*) and basic leucine zipper ATF-like transcription factor 3 (*Batf3*) (13–15, 26, 32, 33). Microglia and CAMs require steady colony stimulating factor 1 receptor (Csf1r) signaling (13). For microglia it was further shown that their dependence on the two ligands, colony stimulating factor 1 (CSF1) and Interleukin-34 (IL-34), seems to differ between brain regions and developmental time points (34). While CSF1 seems to be important for the whole microglial entity during embryonic and fetal development (35–37), gray matter microglia seem to depend more on IL-34 and white matter microglia on CSF1 during postnatal phases and adulthood (34, 38). It remains elusive whether a similar heterogeneity exists for CAMs in the CNS interfaces. In contrast to CAMs, microglia expansion is highly dependent on tumor growth factor β (TGF-β) signaling both during development and maintenance in the adult CNS (26, 39). Another major factor influencing microglial development, maturation and function is the endogenous gut microbiota. Absence of the host microbiota results in pre- and postnatal maturation defects in microglia and further leads to dysfunctional microglia with a hampered immune answer (18, 40). Interestingly, fetal microglia only encounters minor transcriptional changes in the absence of microbiota, whereas the effects become more pronounced in early postnatal and adult microglia (18, 41). Therefore, a detrimental contribution of microbiota to the maturation of regulator networks in microglia upon weaning is suggested. Though, the encounter with pathogenic bacteria and viruses or a dysregulated maternal microbiome during embryonic development can have effects on microglial function later in life. Furthermore, maternal immune activation due to a viral infection during pregnancy can result in microglial pre-priming and a wide spectrum of neuronal abnormalities and phenotypes (42–45).

To protect the CNS during pre- and postnatal development, CNS macrophages are equipped with a wide range of pattern recognition receptors (PRRs) such as Toll-like receptors (TLRs) or nucleotide-binding oligomerization domain-like receptors (NLRs) (46–51). Upon recognition of invading pathogens via PRRs microglia become activated and efficiently remove invading pathogens via phagocytosis (52). This process is accompanied by a release of proinflammatory cytokines and chemokines to activate neighboring microglia but also to recruit other immune cells to the CNS to resolve the infection (53–59). Microglia also upregulate genes involved in the production of inducible nitric oxide synthetase (iNOS) generating reactive oxygen species (ROS) and secrete tumor necrosis factor α (TNF-α), Interleukin-1β (IL-1β) and Interferon γ (IFN-γ) (60, 61). However, these immune defense mechanisms exist together in a fragile balance between fighting off damaging pathogens and causing tissue damage in the developing CNS. This tissue damage is either caused by the invading pathogen itself, but also to a major extent from the immune reaction against the pathogen such as release of ROS, interferons and cytokines which can be neurotoxic, but also T-cell mediated cell lysis, resulting in in neuronal apoptosis, tissue necrosis and CNS malformations (62–64).

In the following, we will explore the current knowledge and literature of different infections occurring during prenatal and early postnatal development of the brain, the responses of microglia and CAMs and their long-lasting implications on brain function.

## Fetal Infections

**Figure 1:**
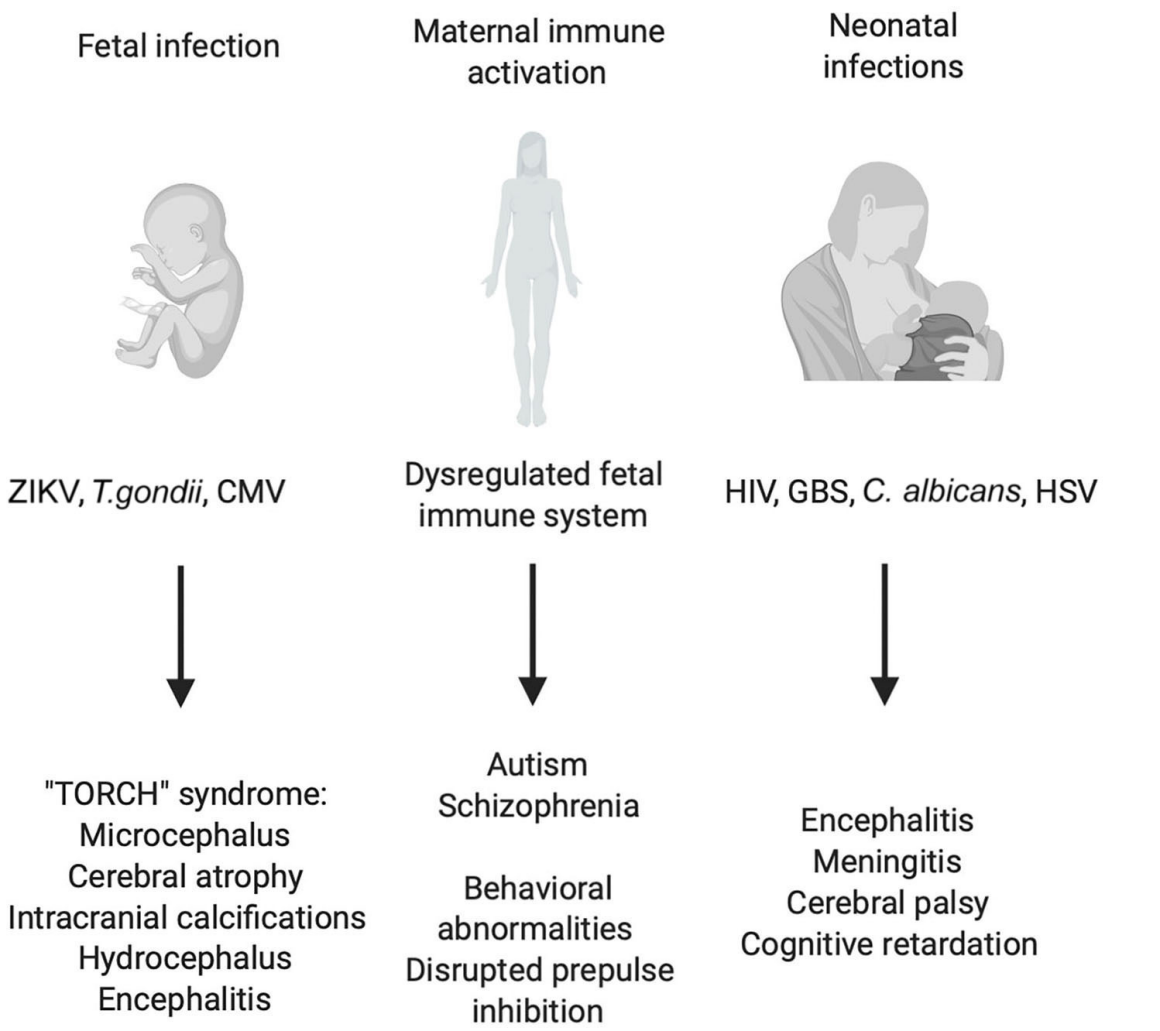
Consequences of early CNS infections and peripheral immune activation for CNS development. Illustration of prenatal and postnatal infections and indirect effects of maternal immune activation (MIA) on the fetus. Different viral, bacterial and parasitic infections can endanger the correct development of the CNS of the fetus leading to a plethora of symptoms grouped under the name “TORCH” syndrome. Also, maternal infection without the transmission of the pathogen to the fetus can have detrimental effects on CNS development which have robustly been linked to schizophrenia and autism spectrum disorder (ASD). Birth and the accompanying contact to environmental pathogens pose a great threat to the unchallenged immune system of the newborn in the worst cases leading to bacterial sepsis, viral encephalitis, and systemic candidiasis.

In a healthy pregnancy the fetus is considered a sterile environment devoid of any living microorganisms under physiological conditions (65). However, upon maternal infection the maternofetal transfer of microorganisms or their by-products can have severe effects on the fetal development (Figure 1). Investigations over the last decades focused on understanding the consequences of these infections on the developing fetal immune system, but also on their effects on the developing CNS (65, 66).

Certain layers of protection are in place to guarantee that no infectious agents are granted access to the developing fetus. The main barrier separating the maternal from the fetal blood circulation is the placenta that develops shortly after conception (67). Beside its barrier function, the placenta links the maternal blood circulation to the embryo to deliver nutrients but also antibodies that protect the developing fetus from blood-borne pathogens via passive immunization (68, 69). In most cases, viral infections of the mother are not transmitted to the fetus (70–72). Besides the physical cell-cell barrier of the placenta, the expression of antiviral interferons (IFNs) produced by the trophoblast and secretion of antimicrobial peptides further inhibits replication and pathogenesis (73, 74). However, infectious pathogens have developed different routes how they can breach this barrier and cause harmful damage to the developing fetus. Maternal infections can be transmitted vertically to the fetus either via the maternal/fetal blood interface (75), the cervical/amniotic sac interface (76, 77) or via transvaginal ascension (78, 79). Mostly at early stages of fetal development during the first and beginning of second trimester, the risk of devastating sequelae is remarkably high mainly due to the critical establishment of the placenta and the beginning of organogenesis.

The most prevalent congenital diseases are caused by a set of microorganisms grouped under the term “TORCH” who manage to overcome the placenta. This acronym stands for *Toxoplasma gondii*, other (*Listeria monocytogenes, Treponema pallidum*, varicella zoster virus, human immunodeficiency virus (HIV), parvovirus B19 and some more), **r**ubella virus, **c**ytomegalovirus and **h**erpes simplex virus 1 and 2 (80). A new member added to this category is the Zika virus mostly due to its recent outbreak in South America (81). TORCH pathologies are grouped because they produce common clinical manifestations like microcephaly, hearing loss, ocular abnormalities and other congenital abnormalities leading in the worst case to fetal loss (Figure 1) (82). The severity of each of these disorders depends on the pathogen and in which gestational period the infection occurs. However, most of these TORCH infections represent with severe CNS infections and malformations (83). How TORCH pathogens are transmitted to the fetus remains predominantly unknown. Addressing the current severe acute respiratory syndrome coronavirus 2 (SARS-CoV-2) pandemic, a recent case study suggested a maternal to fetal transmission in the last trimester of pregnancy. The virus seemed to be transmitted through the placenta since the placental tissue, and both the maternal and fetal blood were positive for the SARS-CoV-2. Interestingly, magnetic resonance imaging showed bilateral gliosis of the white matter of the infant at 11 days of life, but no further long-term deficits (84).

Besides the barrier established by the placenta, the fetus itself has cellular immune protection in place to defend itself against intruders. For a long time, it was thought that the human fetal immune system was immature and will only fully develop during childhood (85, 86). This view has been challenged using new single-cell multi-omic approaches painting a more accurate picture in which both the innate and adaptive arm of the fetal immune system maintain immunity from 4 weeks post conception onwards (87–90).

The mechanism by which maternal infections translate into compromised CNS functionality, malformation and cognitive impairment of the offspring is under extensive research. In the following section we want to take a closer look on the most important TORCH pathogens, their impact on CNS development and the so far known role of the CNS immune system, especially CNS macrophages, on neuropathological outcome after congenital infection.

### Zika Virus (ZIKV)

**Figure 2:**
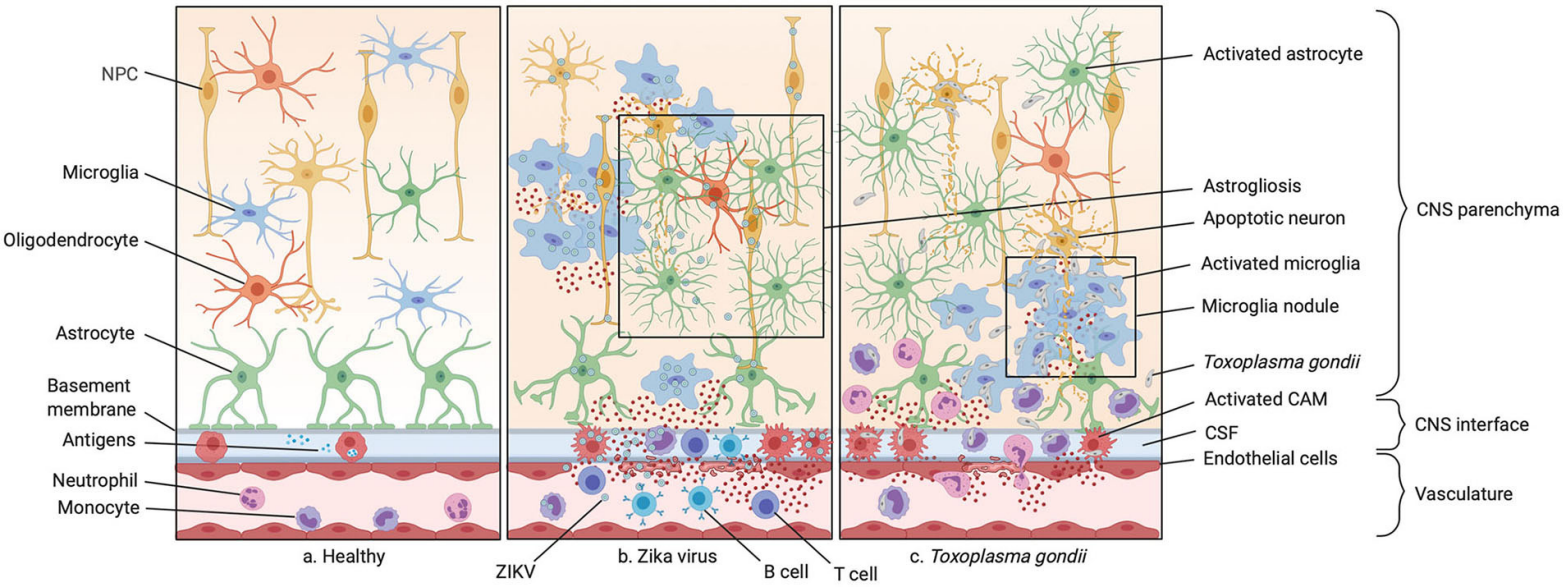
Infections of the prenatal CNS. **(a)** Illustration of the healthy central nervous system (CNS) including the vasculature, the perivascular space, and the brain parenchyma. **(b)** A typical ZIKV infection mostly affecting CNS macrophages inducing neuronal progenitor cell (NPC) apoptosis and recruiting peripheral monocytes and neutrophils to the site of infection via various cytokines and chemokines. The blood-brain barrier (BBB) integrity is compromised in this process. Microgliosis, astrogliosis and microglia nodules around NPC bodies are often observed. **(c)** A typical parasitic CNS infection by *Toxoplasma gondii* is shown. The pathogen gains access to the CNS with the help of circulating monocytes, carrying the parasite to through the compromised BBB, also known as a “Troian horse” infection. This causes microglia nodules, abundant cytokine release by microglia and CNS-associated macrophages (CAMs), and subsequent infiltration by monocytes and granulocytes.

Zika virus (ZIKV) infection during pregnancy has been linked to severe congenital malformations of the CNS (91–93). ZIKV belongs to the family of *Flaviviridae* and was first discovered in Uganda's tropical Zika forest in 1947 (94). The virus is transmitted from mosquitoes of the *Aedes* species to humans which prior to 2007 spared the western hemisphere (95). A widespread outbreak occurred in 2015 in South America, most prominently in Brazil correlating with a strong increase in children born with microcephaly (Table 1) (136). The virus was shown to transmit vertically from the mother to the developing fetus and can then be detected inside the fetal brain (113). ZIKV is able to cross the placenta and infect Hofbauer and cytotrophoblast cells (114–116), however the exact mechanism of transmission is still unknown (76). Infections occurring in the first trimester of pregnancy have the worst consequences for fetal brain development leading to cerebral atrophy and resulting in microcephaly and intracranial microcalcifications (Figure 2) (113, 117, 118). Other infection-related conditions include growth restrictions and ocular abnormalities (137). These symptoms newly added ZIKV onto the list of “TORCH” diseases (76). Additionally, a paraplacental route of infection was described in which the ZIKV was shown to infect the parietal decidua and the amniochorionic membranes (115). Whereas, infections in the first two trimesters cause the above mentioned symptoms, infections during the last trimester do not pose adverse risks for abnormalities in newborns (138, 139). Currently, there are neither vaccines nor therapies against infection of this mosquito-borne disease. It was shown that ZIKV is neurotrophic, meaning that the infection mainly targets the brain (Figure 2) (113). Though some researchers also isolated viral RNA from other fetal organs such as lung, liver, muscle and spleen (114), tissue damage was only observed inside the brain parenchyma (113). Indeed, it was shown that the virus targets neuronal progenitor cells (NPCs), astrocytes and microglia, while only being cytotoxic in NPCs and causing their growth arrest and apoptosis (140, 141). Especially microglia are among the main suspects to disseminate ZIKV in the brain as the particular susceptibility between E6.5-E8.5 in mice coincides with a critical window of microglia development and the beginning of their migration via the newly formed blood circulation toward the brain (142). It was shown that an acute ablation of CSF1R resulting in a depletion of microglia results in a decreased viral load in the brain, supporting a “Trojan horse” model of ZIKV infection (142) (Figure 2). *In vivo* and *in vitro* data underlined that murine microglia progenitor cells from the yolk sac are indeed susceptible to ZIKV infection (142). *In vitro* data showed that infected human induced pluripotent stem cell (iPSC)- derived microglia-like cells co-cultured with neural spheroids leads to propagation of the virus to the neural tissue which supports the claim that microglia act as a viral reservoir for ZIKV and push ahead neural infection in the fetal brain (140). Infected microglia-like cells remained amoeboid with high amounts of phagocytic vesicles compared to uninfected cells taking residency inside the brain spheroid assuming a homeostatic ramified morphology (140). Furthermore, the infected amoeboid microglia only colonized the spheroid underneath the surface and signs of neurodegenerative processes and viral particle release were detected in the spheroids (140). Electrophysiological activity drastically decreased in the infected neuronal spheroids which is not reinstated over time suggesting irreversible neuronal degeneration (140). In line with these results, an *in vivo* study using a mouse model for adult ZIKV infection, showed the presence of the virus in neuronal stem cells in the adult hippocampus leading to the recruitment of IFN-γ producing T cells accompanied by microglial nodules and neuronophagy (143). This specific location together with a resulting reduced Homer-1 expression in synapses let to the speculation that ZIKV infection of hippocampal NPCs leads to cognitive decline in infected adult individuals (143). As already mentioned above, microglia were shown to have an activated phenotype in histological sections of human fetal brains and microglial nodules are described throughout the gray and white matter of the infected fetal CNS (Figure 2) (113). Cultured primary murine microglia showed a proinflammatory phenotype upon ZIKV infection with increased release of IL-6, IL-1β, and TNF-α correlating with an increased neurotoxicity toward fetal NPCs. Culturing of fetal murine NPCs with conditioned media from infected primary cultured microglia revealed a decrease in NPC proliferation and neuronal differentiation (144). These findings could indicate a crucial role of fetal microglial activation and microglial-mediated neurotoxicity during fetal ZIKV infection in humans. A recent study investigated the role of lipid metabolism of ZIKV infected human fetal microglia *in vitro* and linked a higher production of lysophosphatiylcholine (LPC) to the proinflammatory cytokine profile but also to the release of ROS by the infected cells (145). LPC is known to induce inflammasome activation and micro- and astrogliosis. Therefore, the metabolic changes observed in infected microglia could support microglial activation during the infection course (146). However, these findings need further evaluation *in vivo*. Another study performed ZIKV infections in adult macaques revealed a high incidence of neuroinflammation in infected animals and a disruption of the BBB (Figure 2) (147). Here, long-term increased levels of C-X-C motif chemokine ligand 12 (CXCL12), which is a chemokine involved in lymphocyte trafficking and neural repair, were described. Additionally, the authors observed inflammation in the perivascular space and meninges, accompanied by lymphocytic infiltrations (147). This was also reported in fetal human brains, where an infiltration of T and B cells was observed in the perivascular space during ZIKV infection (Figure 2) (113). Lymphocytic infiltrates are observed in neonatal and adult mouse models of ZIKV infections (143, 148), however an infiltration of monocytes or granulocytes could not be observed in infected human brain parenchyma or animal models. In the perivascular space of infected human fetuses, lymphocytic and monocytic accumulations were described and summarized as signs of meningitis. However, it was not further solved if the accumulated mononuclear cells are recruited monocytes or proliferating perivascular macrophages (149). Though, fetal mciroglia seem to play a detrimental role as a reservoir of ZIKV, the viral spread and the neurological symptoms observed, there is so far no data available on the role of fetal CAMs during ZIKV infection.

**Table 1:** Overview of prenatal and postnatal CNS infections.

**Pathogen**	**Critical time for CNS infection**	**Route(s) of infection**	**CNS pathology**	**References**
**Congenital infections**				
*T. gondii*	Third trimester	Transplacental	Microcephaly, hydrocephalus, intracerebral calcifications, epilepsy, intellectual disabilities, encephalitis	(96–99)
*Listeria monocytogenes*	First trimester	Transplacental	Meningoencephalitis, meningitis, hydrocephalus	(99–102)
Varicella zoster virus	Third trimester	Transplacental	Paralysis, seizures, microcephaly, encephaloclastic lesions	(103–105)
Parvovirus B19	Second trimester	Transplacental	Perivascular calcifications, encephalopathy, aseptic menengitis, cerebellar hemorrhage, small cerebellum	(106–108)
Rubella virus	First trimester	Transplacental	Meningoencephalitis correlated with motor and intellectual disabilities, chronic progressive panencephalitis, microcephaly, intracranial calcifications	(109–111)
CMV	Third trimester	Transplacental	Seizures, intellectual disabilities, optic atrophy, sensorineural hearing loss, hydrocephalus, cerebral hypoplasia, periventricular calcifications, cerebral atrophy, porencephaly, delayed myelination	(8, 97, 109, 112)
Zika virus	First and second trimester	Transplacental	Microcephaly, cerebral atrophy, intracranial microcalcifications	(113–118)
**Postnatal infections**				
HIV	Prenatal—breastfeeding	Transplacental, intrapartum, breastfeeding	Microcephaly, developmental delays, focal white matter lesions	(109, 119–121)
HSV	Perinatal	Intrapartum	Encephalitis, aseptic meningitis, microcephaly, hydrocephalus, Encephalomalacia	(109, 122–124)
*Candida albicans*	Perinatal	Nosocomial, intrapartum	Mycotic meningoencephalitis	(125, 126)
GBS	Perinatal	Intrapartum (transvaginal ascension)	Bacterial meningitis, meningoencephalitis, cerebral palsy, cognitive retardation	(78, 127, 128)
Chikungunya virus	Perinatal	Intrapartum	Encephalopathy, meningoencephalitis, microcephaly, cerebral palsy, intraparenchymal hermorrhages	(129–133)
*Escherichia coli*	Perinatal	Intrapartum	Meningitis	(134, 135)

*A variety of pathogens responsible for congenital and postnatal CNS infection, the time in which most of the CNS infections occur, the routes of infection and the CNS pathology is shown*.

### Cytomegalovirus (CMV)

Congenital cytomegalovirus (CMV) infection occurs in roughly 0.2–2.2% of intrauterine infections making it the most common congenital viral infection and the main cause of long-term pediatric conditions (8, 150). CMV belongs to the *betaherpesvirinae*, which is a subfamily of the *herpesviridae* family. After primary infection of a mammal host, the virus persists in a latent state with a low virus titer periodically reactivating throughout life (96, 151). It is transmitted most frequently to pregnant women via smear infection i.e., contact with for example infectious urine and saliva of children (97). 40–50% of pregnant women infected with CMV transmit the virus to the developing fetus (8, 97). CMV is able to cross the placenta and is thus transmitted from the maternal blood to the fetus where it takes residency inside the fetal brain (112). Only 10% of congenitally infected infants are symptomatic (97). However, an infection with CMV is particularly severe during the first and second trimester of pregnancy as it may cause an infection of the trophoblast and interfere with correct development of the placenta often leading to fetal loss (77). Sequelae of congenital CMV infection mainly concern the infant brain and include microcephaly, mental and motor retardation, epilepsy and a progressive loss of vision and hearing (Table 1) (152). Other non CNS-related conditions are hepatosplenomegaly and growth retardation (8, 153, 154). Some studies also proclaimed a link between fetal CMV infection and the development of autism spectrum disorder (98). Like ZIKV, CMV can cross the human placenta thus being transmitted from the maternal to the fetal compartment (77). It is not fully understood how CMV invades the fetus through the placenta, however one study suggests maternal IgG-mediated transcytosis as a potential mechanism (155). The main cellular targets of CMV are brain macrophages, including microglia and CAMs, as well as NPCs in the cerebrum (156). Though, CMV replication has been reported in a wide range of glial cells and neurons in the CNS (157). The number of microglia, perivascular and meningeal macrophages is increased in the murine CMV-infected fetal brains which is not accounted for by proliferation, but rather a recruitment of cells from other areas of the brain as well as transvascular migration from the meninges (156). Even though, it was suggested here that these cells are recruited monocyte-derived macrophages from the fetal liver invading the infected meninges and later the parenchyma (156). Microglial cell numbers increase in infected areas but also in regions where the viral antigens are not detected (156). Interestingly, choroid plexus macrophages were also described to be elevated in numbers in this murine model for fetal CMV infection (156). As also reported during fetal ZIKV infection, microglia assemble in nodules in infected CMV foci in the brain tissue (156, 158, 159). They are located around degenerating CMV-infected neurons engaging in neuronophagy and clearance of dead cells (158, 159). *In vitro* studies of human fetal microglia and astrocytes showed that CMV-infected microglia can secrete TNF-α, IL-1β, and IL-6 orchestrating an antiviral response and dampening viral replication for example in infected astrocytes (55). This study suggested that CMV-infected fetal astrocytes are not competent in mounting an antiviral response but rather secrete cytokines such as CC-chemokine ligand (CCL2) to recruit microglia (55). *In vitro* infection of a murine microglial cell line with CMV showed extensive metabolic alteration with a shift of microglia metabolism toward glycolysis supporting the proinflammatory phenotype and activation previously reported upon CMV infection (160). Microglia and CAMs are a cellular target of CMV and are associated with neuronal degeneration and tissue damage at infectious foci in the brain. Therefore, further investigations should be performed if these CNS immune cell populations are potential therapeutic targets during fetal CMV infections. First results already suggested that targeting fetal microglia and CAMs during CMV infection in rats by intracranioventricular injection of depleting clodronate liposomes resulted in an improvement of survival, neuropathology and cognitive functions in the born litter (161).

### *Toxoplasma gondii* (*T. gondii*)

The obligate intracellular protozoan parasite *Toxoplasma gondii* (*T. gondii*) causes one of the most prevalent chronic infections with around 30% of the world population being infected (162, 163). The only known sexual stages of the *T. gondii* life cycle take place in the family of the *felidae* (164). Here oocysts or sporozoites are formed and excreted in cat feces (165). Ingestion of oocysts by intermediate hosts such as rodents and birds (166) lead to a transformation into rapidly proliferating tachyzoites which are able to spread into different tissues via the blood stream, infected all nucleated resident cells and cause host cell lysis (167). Within the host cells tachyzoites replicate in specialized parasitophorous vacuoles escaping lysosomal fusion, finally induce lysis of the host cell and disseminate adjacent cells (168). Once they arrive in tissues like the CNS or muscle, they convert into bradyzoites or tissue cysts, allowing them to evade from the immune system and persist for many years (165). *T. gondii* can be transmitted into humans through consumption of contaminated food or water with cat fecal oocysts (169). Alternatively, oocysts can be ingested by livestock forming tissue cysts which are then transmitted into humans by consumption of uncooked contaminated tissue (165). In rarer cases *T. gondii* is transmitted via blood transfusion or organ transplantation (170, 171). *T. gondii* infection in immunocompetent adult individuals is asymptomatic or causes only mild flu-like symptoms in most cases, but opportunistic reactivation from cysts into tachyzoites can occur in immunosuppressed individuals, such as HIV patients, leading to a toxoplasmic encephalitis in adults (Table 1) (172, 173). However, vertical transmission of *T. gondii* from the mother to the fetus can be harmful or even fatal. Though, maternal to fetal transmission only occurs when the mother is primary infected during or up to 10 weeks before pregnancy (174). While infection in early pregnancy poses a small risk to fetal transmission (less than 6%), rates of transmission increase up to 81% in the third trimester (175). This was assumed to be due to a differential expression of placental TLRs during pregnancy (176). However, the consequences of fetal *T. gondii* infection are highest in early embryos and may result in severe congenital toxoplasmosis with CNS pathologies, including microcephaly, hydrocephalus, intracerebral calcifications, epilepsy, intellectual disabilities or even spontaneous abortion and fetal death (Table 1) (175, 177–181). Fetuses infected in late gestation are born normal, but can develop CNS symptoms and retinochoroiditis after birth (175). Studies suggest that maternal-fetal transmission starts with initial infection of the uterus, followed by the extravillous trophoblasts of the placenta as the parasite moves from cell to cell, and eventually lead to the infection of the fetus (182). Similar to the adult infections, it is assumed that *T. gondii* infects circulating cells of the developing fetus such as macrophages and use these cells as a “Trojan horse” to gain access to immune privileged sites such as the CNS or reaches the CNS via paracellular and transcellular infection routes (Figure 2) (183–187). *T. gondii* can infect almost any nucleated cell, but chronic infection seems to be neurotropic in a sense, since it is cleared in other tissues over time (except musculature) (172, 188). While neurons, astrocytes and microglia are infected during the acute phase by tachyzoites, it seems that bradyzoite cysts develop mostly in neurons as shown in humans and mice (172, 189, 190). *In vitro* studies with rat CNS cells showed that 10% of neurons and 30% of microglia were infected with *T. gondii* (191). However, 93% of the parasitophorous vacuoles only contained one to two degenerated parasites in microglia, while neurons contained up to 8 proliferating parasites and most of the bradyzoites four days after acute infection (189). During congenital toxoplasmosis, multifocal diffuse tissue necrosis in the developing brain is mainly caused by parasite induced lysis of infected neurons (192). Close to these lesion sites, accumulation of microglial nodules have been described (Figure 2) (192). Most of the studies investigating the role of microglia and CAMs during *T. gondii* infection focused on studies of the adult CNS, whereas only less information is so far available on their role during congenital toxoplasmosis. In the adult CNS, microglia and infiltrating CD11b^+^ CD45^high^ monocyte-derived macrophages are one of the main source of IFN-γ during *T. gondii* infection, which is critical to control parasitic spread in acute but also chronic infection (193). One study showed that 37% of the IFN-γ positive cells were CD11b^+^CD45^low^ microglia while 63% were CD11b^+^CD45^high^ positive cells, most likely monocyte-derived macrophages (193). Recruited Ly6C^high^ monocytes, but also infiltrating Ly6G^+^ granulocytes are a detrimental source of IFN-γ (194). Deficiency of IFN-γ upon *T. gondii* encephalitis in adult mice resulted in a reduced chemokine and cytokine release in the CNS parenchyma with a decrease in leukocyte infiltration to the infected CNS tissues (195). Beside IFN-γ, several more effector molecules such as nitric oxide (NO), TNF-α and IL-12 were shown to be involved in the inhibition of parasitic spread in the adult CNS (196, 197). Besides the upregulation of effector molecules, microglia exhibit a hypermigratory phenotype upon *T. gondii* infection (198). A recent study reported hypermigration of primary murine neonatal cortical microglia through signaling of the neurotransmitter γ-aminobutyric acid (GABA) after *T. gondii* infection, which was not visible after lipopolysaccharides (LPS) or heat-inactivated *T. gondii* stimulation, and inhibition of GABA synthesis, receptors and regulators led to an inhibition of the hypermotility of microglia (199). To this day, data about disease mechanisms of congenital *T. gondii* infection remains scarce and there is an urgent need to address the role of microglia and CAMs in congenital toxoplasmosis. The neurotoxic properties of *T. gondii* and the resulting microglial activation are likely to cause malformation of the neuronal network in the developing fetal CNS, resulting in the typical neurodegenerative symptoms of the prenatally acquired disease. Therefore, understanding microglial involvement in the disease manifestation during fetal development could be key for new treatment paradigms.

## Maternal Immune Activation (MIA)

There is increasing evidence that maternal infection and consequential immune activation during pregnancy can have detrimental effects on fetal brain development and cause morbidity even without transmitting the pathogen to the fetus (200, 201). Recently, maternal immune activation (MIA) was implicated in long-term changes within the CNS which can affect CNS function much later in life. It is not entirely solved yet what the molecular mechanisms are for these long-term effects on the CNS, but the spotlight is turned on a detrimental involvement of the immune system (202). Most of all, schizophrenia and autism spectrum disorders (ASD) have been linked to a dysregulated fetal immune system which can induce changes during CNS development and maturation and many studies suggest that this is traced back to an initial activation in the womb due to non-transmitted maternal infection during pregnancy (Figure 1) (203). Elevated levels of serum proinflammatory cytokines in the infected mother may be harmful for placenta barrier integrity and lead to increased induction of cytokine levels in the fetus (204). It is thought that an overexpression of proinflammatory molecules such as IL-6, TNF-α, and IL-1β, which are required for neurodevelopment under physiological circumstances (205), can interfere with correct development of the neuronal network (206). Studies indicate that elevated maternal cytokines such as IL-8 or TNF-α correlate with a higher incidence of schizophrenia in their uninfected offspring (Figure 1) (207, 208). In many studies, absence of fetal infection is rarely confirmed, making it more difficult to assess whether long-term neurological differences are due to indirect effects of maternal inflammation or direct cytopathic effects in the fetus. To investigate the causal mechanisms behind MIA and immune dysregulation in the fetus, researchers have developed several rodent MIA models where the maternal immune system is manipulated by injections of poly-(I:C) or LPS to mimic viral or bacterial infections in the mother. Injection of poly-(I:C) into pregnant females was shown to cause disrupted prepulse inhibition in the offspring, similar to what was observed in schizophrenic patients (209). Injection of LPS in pregnant mice caused behavioral changes (210), altered synaptic pruning (211) and structural changes in the hippocampus leading to impaired memory and learning abilities (212) in their offspring (Figure 1). IL-6 was shown to be elevated after maternal immune activation both in maternal serum and fetal tissue (209). Injection of recombinant IL-6 or antibody mediated depletion of IL-6 demonstrated that elevated IL-6 levels were in part responsible for the exploratory, social, and other behavioral abnormalities observed in the offspring of poly-(I:C) injected pregnant mice (209). In line with these findings in poly-(I:C) treated pregnant mice, injection of LPS into the mother also led to increased IL-6 expression in the fetal brain (212). Beside IL-6, IL-1β, and IL-10 levels were also increased following poly-(I:C) injection into pregnant mice (213–215). Elevated CXCL-8 in maternal serum was shown to induce neuroanatomical alterations in the offspring (216). Interestingly, these regions often coincide with regions shown to be dysregulated in schizophrenia patients such as parahippocampal and superior temporal gyrus volume reductions (216). Injection of LPS into pregnant mice induced elevated levels of TNF-α in the fetal brain (217). It seems that fetal microglia are primed by MIA via potential epigenetic imprints leading to a magnified immune response later in life upon a secondary challenge (43, 218, 219). MIA offspring that received an LPS injection later in adulthood displayed an increase in IL-1β in the hippocampus (220). However, this effect was brain region specific (220). It was shown that microglia activation steadily increases throughout early life, peaking during adolescent which coincides with behavioral abnormalities also observed in schizophrenia patients (221). Many studies report an elevated number of reactive amoeboid microglia expressing high levels of major histocompatibility complex (MHC) class II and CD68 in the fetal and neonate brain indicating an activated status after injection of poly-(I:C) or LPS into pregnant mice (222–225). However, these results are highly debated as many groups fail to confirm elevated microglia numbers (226), nor do they report the presence of activated Mac-2 expressing microglia in fetal CNS parenchyma after poly-(I:C) injections (227). The same discrepancy is also present in *post-mortem* studies quantifying microglia numbers in the brain of diseased schizophrenia patients (228). Microglia in MIA offspring were shown to display different migratory dynamics as tangential and radial spreading to the hippocampus, corpus callosum, striatum and somatosensory cortex seems to be delayed (229). This in turn could alter developmental processes where microglia involvement is required (229). Even though epidemiological studies linking MIA during pregnancy to neuropsychiatric diseases in the offspring are still sparse, newly developed animal models start to shed light on the role of microglia in this process while the role of CAMs remains to be explored.

## Postnatal Infections

Birth is the first direct contact of the newborn with the outside environment including a plethora of different microbes (230). In general, the human body lives in symbiosis with many different microbes including the species colonizing our gut and skin. The mucosa of the female genital tract is inhabited by a variety of commensal bacteria and fungi unique to every woman and vitally important for her health (231). However, beside the beneficial microbiota the human body is constantly eradicating potentially harmful pathogens which can enter the body via different routes or co-colonize microbiotic niches such as the mucosa of gut, lung or the reproductive tract. During birth, these mucosal organisms can come into contact with the newborn and its unchallenged immune system. With that said, the intrapartum period is a critical time for microbiotic colonization but also where serious infections may occur (Table 1) (232, 233). Transmission of pathogenic microorganisms from the maternal vaginal tract to the newborn can lead to dissemination to several organs of the infant, possibly resulting in long lasting morbidities or even death (Table 1) (234, 235). After birth, the infant body is immediately in contact with different microorganisms either beneficial or pathogenic. Therefore, the breastfeeding period plays a major role in preventing early diseases since it not only includes nutrients, enzymes, antimicrobial proteins/peptides, growth factors, antioxidants, anti-inflammatory elements but also transfers maternal immunoglobulins through the breast milk to compensate for the lack of immune memory in the newborn (236–239). Furthermore, studies showed that human breast milk facilitates the establishment of the neonatal microbiota indicating long term benefits for the infant beyond the breastfeeding period (239, 240). To further protect the newborn in this critical time the WHO recommended vaccination as soon as possible after birth, optimally in the first 24 h to guarantee protection against polio, hepatitis B and tuberculosis. However, vaccinations cannot establish immunity against infections occurring shortly after birth and many infections occurring in the first few weeks do not have a vaccine yet (241). Accompanied by profound changes of an immune system which needs to be fine-tuned to guarantee a balance between tolerance and immunity, the encounter of a vast assortment of antigens pose a high risk for infections to occur (Table 1) (242, 243). Studies have shown that the adaptive arm of the immune system presents certain differences compared to later time points in life (244–247). One interesting divergence is the shifted T helper (Th) cell balance in favor of a Th2 cell response during fetal and early postnatal development (248, 249). This in turn inhibits Th1 and Th17 response backing tolerance to the mother, however, making the newborn more susceptible to bacterial and fungal infections (248, 249). Furthermore, it was shown that suppressive regulatory T cells and impaired antigen-presenting cells in the fetus increased disease susceptibility at birth and may hinder effective early vaccination (244–247). As mentioned above, this was a long time assumed to point toward an immature immune system, which was recently revoked by several multi-omics studies (87–90). The maturation of the resident immune cells of the CNS was only recently started to be explored during postnatal stages using bulk and single cell RNA-sequencing (250–252). However, it is clear that CAMs and microglia play important homeostatic functions during early postnatal phases (253). In the following we will discuss how the most prevalent neonatal infections are able to establish CNS infection and explore the role of CNS macrophages in this context and the far-reaching consequences for the developing infant.

### Human Immunodeficiency Virus (HIV)

**Figure 3:**
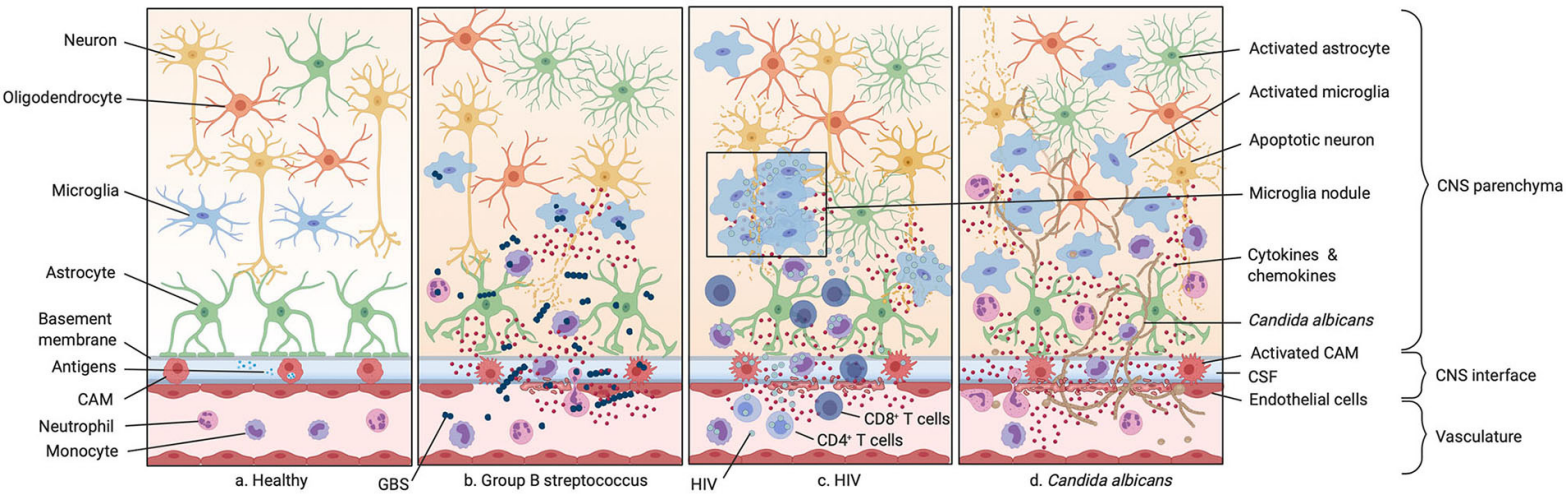
Infections of the neonatal CNS. **(a)** Illustration of the healthy CNS including the vasculature, the perivascular space, and the brain parenchyma. **(b)** Group B streptococcus (GBS) infection. GBS enters the CNS either paracellularly, via transcytosis or exploiting trafficking phagocytic host cells. This leads to the secretion of inflammatory cytokines by resident macrophages in the perivascular space and meninges. As GBS spreads more into the CNS this inflammatory response is also mounted by endothelial cells and microglia, leading to leakage of the BBB and therefore an increase number of monocytes and neutrophils in the CNS. **(c)** HIV infection. HIV infects CD4^+^ T cells, monocytes, and macrophages. Infection of circulating monocytes helps HIV to enter the CNS where it infects other tissue-resident macrophages. This leads to an acute immune response with secretion of proinflammatory cytokines. **(d)** *Candida albicans* infection. *Candida albicans* enters the CNS either para- or intracellularly or shuttled inside circulating monocytes. Infection of the CNS leads to an activation of microglia, astrocytes, and oligodendrocytes resulting in the secretion of cytokines and chemokines and subsequently to the recruitment of neutrophils and other circulating monocytes.

Human immunodeficiency virus (HIV) is a lentivirus and belongs to the family of the *retroviridae* (254). Retroviruses are single-stranded RNA viruses that are able to integrate their genome after reverse transcription into the hosts DNA and thereby induce a latent infection which can persist for several decades (255). Infected cells convert the viral RNA into double-stranded DNA by using an enzyme called reverse transcriptase which is then incorporated into the hosts genome with a viral integrase and other host co-factors (256). There are two different types of HIV, HIV-1 and HIV-2, with the first being responsible for most of the global HIV infections (255). HIV is transmitted by sexual contact, exposure to infected body fluids and from mother to child during pregnancy, birth, and breastfeeding (109). The risk of vertical transmission decreased dramatically after the development of antiretroviral therapy (ART) during pregnancy and breastfeeding period for infected women in developed countries (257, 258). Primary infant infection is mostly asymptomatic, but disease progression is significantly faster than in adults (259). Before the introduction of ART, half of the infected children developed progressive HIV-1 encephalopathy (PHE) resulting in microcephaly, developmental delays, and focal white matter lesions (Table 1) (119–121). PHE further manifests in basal ganglia calcification and white matter lesions (Table 1) (260). In PHE lesions, microglial cells were often found to accumulate in nodules and seem to be the major cell type infected with the virus. Perivascular lesions with infected proliferating microglia and infiltration of CD8^+^ T cells and monocytes were further seen in children with HIV encephalitis (Figure 3) (261). Though, chronic neurological impairment is still obtained in HIV-1 seropositive children under ART (262–264). HIV is mostly known for infecting peripheral helper T cells via the cluster of differentiation 4 (CD4) receptor and either C-X-C chemokine receptor type 4 (CXCR4) or C-C chemokine receptor type 5 (CCR5) as a co-receptor, leading to a loss of these cells during acute infection (265). However, CD4^+^ T cell numbers recover after acute infection but decrease in the course of years, ultimately leading to the development of the acquired immunodeficiency syndrome (AIDS) which is characterized by low T cell counts in the blood and the occurrence of rare opportunistic diseases (266, 267). Tissue resident macrophages across different organs and circulating monocytes are also used as a viral reservoir, since they also express low levels of CD4 and the co-receptors CXCR4, CCR5 and also CCR3 in infants and adults (268). Despite CXCR4 expression macrophage-tropic (M-tropic) HIV variants mostly use CCR5 as a co-receptor to enter the cell. These viruses can bind CD4 more efficient and show enhanced interactions of their envelope glycoprotein 120 (gp120) with CCR5 (269–271). Especially yolk sac-derived tissue-resident macrophages represent an ideal viral reservoir because they are long-lived and self-renewing, making them a perfect target for latent infection (272). Yet the role of macrophages as long-term reservoirs for replication-competent viruses remained controversial. A recent study showed the presence of macrophage reservoirs in the brain of simian immunodeficiency virus (SIV) infected macaques, which is the equivalent to HIV in humans (272). Perivascular macrophages and parenchymal microglia were shown to be infected during acute SIV infection in adult macaques and acute HIV infection in adult humans (273–275). Furthermore, human fetal microglia can be efficiently infected with HIV-1 in *ex vivo* cultures (276). Data suggested that HIV reaches the brain via infection of bone marrow-derived monocytes which are visible in the CNS during acute infection and through the cerebrospinal fluid (CSF), using them as a “Trojan horse” similarly as described for *T. gondii* infection (Figure 3) (277–280). Release of proinflammatory cytokines and ROS from infected microglia, as well as synthesized viral molecules such as gp120 and trans-activator of transcription (Tat) can lead to neurotoxicity and neural injury (264, 281–283). A new mechanism of microglial activation was described for neonatal microglia *in vitro* where exposure to viral Tat resulted in the downregulation of microRNA-124 (miRNA-124), leading to increased methyl CpG binding protein 2 (MECP2) and signal transducer and activator of transcription 3 (STAT3) expression (284). Tat-mediated neonatal microglial activation was also induced by upregulation of miRNA-34a leading to the downregulation of NOD-like receptor C5 (NLRC5), which is a negative regulator of NF-κB signaling, and by NLR family pyrin domain containing 3 (NLRP3) inflammasome activation, both leading to enhanced cytokine release including IL-6 and IL-1β (285, 286). Primary human microglial activation was further shown by the upregulation of pro-inflammatory molecules upon HIV infection such as IL-8, IL-6 CCL2, TNF-α, CCL5 and activation of IL-1β and caspase-1 (287, 288). In adult patients but also in young infants, constant microglial activation is implicated in HIV-associated neurocognitive disorders (HAND), such as HIV-associated dementia (HAD) (289, 290). This correlates with elevated levels of TNF-α, IL-1β, caspase-1 and iNOS in microglia of HAD patients (291–293). In adult patients with HIV encephalitis, perivascular infiltration of CD14^+^ CD16^+^ HIV-infected monocytes was also correlated with the onset of dementia (290). A recent study showed that other human macrophage populations from lung and abdomen are able to restrict viral spread via sterile alpha motif and histidine/aspartic acid domain-containing protein 1 (SAMHD1) effectively compared to human microglia, which express similar levels of SAMHD1, but are more susceptible to HIV-1 infection (294). Even in the absence of neurological symptoms, HIV infected patients showed signs of immune activation in the CNS. Obtained microglial activation markers include MHC class II, CD163, IL-1β, and TNF-α levels in the cerebral cortex and white matter of seropositive patients (295–297) and TNF-α, β2-microglobulin and neopterin in the CSF, which are further markers of microglia as well as CAM activation (295). Overall, data suggest that microglia and CAMs at the CNS interfaces can serve as a potential reservoir for HIV even in infants and viral-induced constant microglial activation leading to neurodevelopmental impairment in children and potential onset of neurodegenerative disease later in age.

### Herpes Simplex Virus (HSV)

Herpes simplex viruses (HSV) belong to the family of the *herpesviridae* and are split into two different serotypes: HSV-1 and HSV-2 (298). Like all herpesviruses they contain double-stranded DNA and are able to cause a latent infection (298). HSV infections, if symptomatic, often manifests in oral or genital lesions (298). Both of these viruses are, together with varicella zoster virus, the only herpesviruses that are neurotropic and establish a latent state in dorsal root ganglia (299). Therefore, HSV infection can also occur in the CNS, leading to more severe outcomes such as herpes simplex encephalitis (HSE) and aseptic meningitis (Table 1) (122). Interestingly, HSV-1 is the cause of 90% of HSE in adults, while HSV-2 is mostly associated with neonatal HSE and immunocompromised patients (300). Neonatal herpes is rare but fatal in 60% of cases if not treated (301). Even with treatment, neonatal infection of the CNS can still be fatal and leads to moderate-to-severe neurological abnormalities in more than 50% of the cases (Table 1) (123, 302). Human fetal astrocytes and neurons have been shown to be efficiently infected *in vitro* by HSV-1, but do not show an induction of cytokine and chemokine expression upon infection (303). Fetal human microglia, however, did not support viral replication but showed an extensive induction of proinflammatory factors such as CXCL10, TNF-α, CCL5, or IL-1β (303). Interestingly, CXCL10 was found to reduce viral replication in neurons, indicating a protective role of microglial-derived CXCL10 (303). Therefore, microglia could play an essential role in controlling HSV-1 replication also in the neonatal brain, as also indicated by other studies during adult encephalitis (304, 305). Upregulation of glutamate transporter 1 (GLT-1) and glutathione was found in microglia, which is an antioxidant with antiviral properties (306). Additionally, IL-6 produced by microglia was shown to protect against neuronal loss after HSV-1 infection (307) underlining the importance of microglia in this context. Purinergic receptor P2Y12^+^ microglia processes extended to HSV-1 positive neurons in adult HSE in humans and were shown to accumulate in an amoeboid shape around them (308). Extensive cytokine and chemokine expression of cultured human microglia such as TNF-α, IL-1β, IL-10, IFN-α/β/γ and CXCL10, CCL2, CCL4 and CCL5 was observed during HSV-1 infection and upregulation of these effector molecules is partially mediated by TLR2 (303, 309). Higher expression of CXCL10, CCL2, and CXCL9 was also found in infected BALB/c mice (310). TLR3, which recognizes double-stranded viral RNA, was also shown to be highly relevant in fighting HSV infection as two otherwise healthy children with a dominant-negative TLR3 allele were reported with HSE (311). More cases of children with HSE were found with deficiencies in proteins involved in the TLR3-signaling pathway such as TNF receptor asscociated factor 3 (TRAF3), TIR-domain-containing adapter-inducing interferon-β (TRIF) and Unc-93 homolog B (UNC93B) (312–315). Yet, an involvement of microglia and CAMs, which do express high levels of TLR3, needs to be elucidated in this context (316, 317). A recent study, however, demonstrated that adult microglial type I IFN production is dependent on stimulator of interferon genes (STING) and cyclic GMP-AMP synthase (cGAS) (305). Adult mice deficient in cGAS and STING were shown to be susceptible to HSE (305). STING deficiency in isolated neonatal microglia was shown to lead to a higher viral load in the cells and microglial type I interferons production is dependent on STING signaling (305). In conclusion, neonatal HSV infection of the CNS is still a threat and more research is needed to balance a strong immune response restricting viral spread and constant neuroinflammation damaging the developing CNS.

### Candida albicans

Fungal infections pose a serious threat to nurseries and intensive care units as newborns, especially premature ones, seem to be most at risk (318). *Candida albicans* is the most prevalent fungus inhabiting the oropharynx and genital tract of humans and is part of the commensal gut microbiota of 60% of the population (319, 320). At the same time, it is also the major cause of fungal related pathology (Table 1) (319). Opportunistic infections can become rampant if the immune system is disturbed or suppressed for example during pharmacological treatments or immunodeficiency diseases resulting in systemic candidiasis (321). This puts mostly preterm newborns at risk, presenting with an unchallenged immune system and often undergoing heavy antibiotic or corticosteroid treatments, the latter destined to support correct development of the premature lung (322, 323). *C. albicans* is transmitted to the fetus *in utero* and to the newborn either nosocomially (in the hospital), or during birth (125, 126). Typically, mucosal membrane colonization is an important event to protect the healthy newborn from invasive candidiasis, while leaving an incidence of 0.5–20% of newborns in the US which are still developing systemic candidiasis (Table 1) (324). Another risk factor for developing candidiasis is necrotizing enterocolitis which was shown to facilitate fungal invasion into the blood stream (325). The mortality rate of newborns suffering from an acute candida infection is a staggering 35–40% despite therapy (325). Involvement of the CNS constitutes a major complication and occurs in more than half of fungal infections with conditions like brain abscess and mycotic meningoencephalitis (326, 327). Although the mechanism of how *C. albicans* breach the BBB is unclear, it is believed that it can either cross para- or intracellularly or shuttled inside circulating monocytes (328, 329). *C. albicans* produces a toxin called candidalysin which was shown to activate microglia, astrocytes and oligodendrocytes (330). Furthermore, microglia do express TLR2, Dectin-1 and complement receptor 3 which are essential to recognize *invading C. albicans* in the CNS (328, 330). Studies in adult mice indicate that microglia together with astrocytes and oligodendrocytes secrete CXCL1 and CXCL2 which induces neutrophil recruitment to the brain and essentially supports clearance of the pathogen from the brain (Figure 3) (330). It is not clear whether the same interplay of glial cells and neutrophils takes effect in newborns. An interesting parallel is that neutrophils isolated from newborns have decreased chemotactic capabilities compared to adults (331). Neutropenia in the CSF has been observed in certain cases of newborn CNS candidiasis (324, 326). It is unknown whether microglia display any deficiencies explaining the grim outcome of candidiasis affecting the CNS. This interplay of glial cells and neutrophils was shown to be crucial for containment and efficient clearance of the pathogen as patients with neutropenia suffered worse outcomes of the infection. This interplay is essentially dependent on the downstream pattern recognition receptor adaptor molecule C-type lectin receptor–Syk adaptor (CARD9) (330). The secreted toxin candidalysin induced microglia to release IL-1β and CXCL2 which in turn resulted in the efficient recruitment of neutrophils to the CNS (Figure 3) (330). Furthermore, microglia recruited Ly6C^+^ monocytes to the infected CNS which can further contribute to the recruitment of neutrophils (Figure 3). Specific deletion of *Card9* in microglia resulted in an impaired neutrophil recruitment and might trigger fungal susceptibility (330). In line with these findings, it was shown that young patients with a defect in the *Card9* gene cannot produce neutrophil targeted chemokines and suffer from uncontrolled fungal growth and primary immunodeficiency (330). Recruited neutrophils start secreting chemokines themselves, amplifying this positive feedback loop and driving inflammation of the CNS (332). Interestingly, *Card9* deficiency did not result in candida invasion of other organs like the spleen, liver or kidney (333). It is currently unknown whether the CARD9 adaptor protein is also important for other microglia functions like phagocytosing or killing yeast cells. This detrimental role of microglia in the recruitment of neutrophils suggest a central role in the immune answer against *C. albicans* infection in infants and might also be a key target in understanding neonatal susceptibility to *C. albicans* infections. So far there is only limited data on the specific role of CAMs in neonatal *C. albicans* infections available.

### Group B Streptococcus (GBS)

Group B streptococcus (GBS) or *Streptococcus agalactiae* is a gram-positive encapsulated bacterium. Infection with GBS is particularly dangerous for newborns presenting a high rate of morbidity of infected individuals who survive the primary neonatal infection (334). GBS is a major, potentially lethal cause of bacterial meningitis in newborns which is an infection of the CNS membranes covering brain and spinal cord (Table 1) (335–337). Affected newborns most frequently suffer from neurological consequences such as cerebral palsy, cognitive retardation, loss of vision and hearing and seizures (Table 1) (127). GBS naturally colonizes the genital tract of 1 in 4 healthy women, only in a minority of cases leading to an ascending *in utero* infection of the neonate via the amniotic fluid or during delivery via aspiration of vaginal fluids (338, 339). However, GBS is the leading cause of neonatal sepsis with an occurrence of around 42 cases per 100.000 live births and a mortality rate of around 9% (340, 341). Sepsis is divided into early onset sepsis if it occurs within the first 7 days of life and late onset sepsis if it occurs until 3 months after birth (341). Late onset sepsis often leads to meningoencephalitis which has a mortality rate currently of around 8.3% (Table 1) (340). Here, an intestinal route of GBS infection was suggested in neonates (337). Often GBS enter via the microvasculature in the choroid plexus where they adhere to endothelial cells and are able to cross the BBB either paracellularly, via transcytosis or exploiting trafficking phagocytic host cells (342, 343). In the interfaces of the brain, they encounter, besides recruited circulating monocytes, also the resident perivascular and meningeal macrophages (337). It was shown that perivascular and meningeal macrophages play an important role during bacterial infection of the CNS by secreting pro-inflammatory cytokines such as IL-1β, TNF-α, and IL-6 and phagocytose invading bacteria (Figure 3) (344). A robust inflammation is mounted by both endothelial cells outside the brain, as well as CAMs inside the brain facilitating the entry of more pathogens through the now leaky BBB and increasing the number of emigrating Ly6C^high^ monocytes and neutrophils at the site of infection (Figure 3) (345). *In vitro* studies have shown that microglia react to GBS by TLR2/Myeloid differentiation primary response 88 (MyD88) dependent pathways leading to NO secretion specifically inducing apoptosis in neurons (346). Newborns consequently develop hydrocephalus, ischemia and increased intracerebral pressure and brain injury leading to more neuronal apoptosis (Table 1) (347). In a mouse model of vaginally colonized pregnant females, the authors were able to recapitulate most of the hallmarks of neonatal infection with GBS and following meningitis (348). Immunofluorescent imaging of infected neonatal mouse brains confirmed an activated amoeboid phenotype of microglia (348). However, in this new model of vaginal infection with GBS of pregnant mothers, the authors did not observe an increase in inflammatory cytokines when comparing infected and uninfected pups indicating that neuronal damage is rather induced by the secretion of ROS (348). In contrast, studies performed in GBS infected adult mice attribute a high importance to IL-1β signaling as they show that IL-1β deficient mice cannot keep GBS infection from disseminating to target organs such as the brain and leading to death (349). Long term sequelae of pups infected with GBS indicate a decrease in exploratory behavior in these animals impaired learning and memory and decreased glutamate levels (348). This is in line with the observation that over 30% of children that survived GBS meningitis suffer from neurodevelopmental impairment (128). As previously suggested, the exact role of microglia and CAMs in GBS mediated meningoencephalitis remains ill-defined and needs to be further explored (337).

## Concluding Remarks

Besides the many improvements in hygiene and preventive treatments in recent decades, CNS infections are still a threat for the developing fetus and newborn. Information about pathogen-specific infection mechanisms remains scarce. This is due to the limited accessibility of human samples, however the refinement of *in utero* animal infection models will help to further investigate this issue in the future. A growing body of evidence implicates that especially microglia are an important factor for the outcome of such infections. Their crucial role in CNS development and their interplay with all different cells in the CNS, which is required for the establishment of a functional neuronal network and maintaining CNS homeostasis, may be disrupted in the presence of a pathogen. Whether tissue damage is directly caused by the necrotic properties of pathogens or through the constant activation of CNS macrophages and the subsequent secretion of proinflammatory molecules resulting in long-term complications, is dependent on the respective type of infection and needs to be studied accordingly. The embryonic origin of CAMs has only recently been revealed. Thus, prior studies did not separate long-lived CAMs from infiltrating monocytes in the context of an infection. However, CAMs should not be neglected since they are the “gatekeeper” in the interfaces of the brain and come in contact with intruders earlier than microglia. Due to the longevity of CAMs and microglia a potential role as viral reservoirs needs to be considered and was implicated in recent studies. In conclusion, understanding the impact of prenatal and neonatal CNS infection on the development and the functionality of CNS macrophages may help to comprehend the underlying mechanisms leading to pathogenesis and long-term sequelae of the infant.

## Author Contributions

AO and PP wrote the manuscript and designed the figures. KK and DE conceptualized, supervised, and edited the manuscript. All authors listed approved it for publication.

## Conflict of Interest

The authors declare that the research was conducted in the absence of any commercial or financial relationships that could be construed as a potential conflict of interest.
